# Distinct microbial populations are tightly linked to the profile of dissolved iron in the methanic sediments of the Helgoland mud area, North Sea

**DOI:** 10.3389/fmicb.2015.00365

**Published:** 2015-05-01

**Authors:** Oluwatobi Oni, Tetsuro Miyatake, Sabine Kasten, Tim Richter-Heitmann, David Fischer, Laura Wagenknecht, Ajinkya Kulkarni, Mathias Blumers, Sergii I. Shylin, Vadim Ksenofontov, Benilde F. O. Costa, Göstar Klingelhöfer, Michael W. Friedrich

**Affiliations:** ^1^Microbial Ecophysiology Group, Faculty of Biology/Chemistry, University of BremenBremen, Germany; ^2^MARUM, Center for Marine Environmental Sciences, University of BremenBremen, Germany; ^3^Alfred Wegener Institute, Helmholtz Centre for Polar and Marine ResearchBremerhaven, Germany; ^4^Institute for Inorganic and Analytical Chemistry, Johannes Gutenberg UniversityMainz, Germany; ^5^Department of Chemistry, Taras Shevchenko National University of KyivKyiv, Ukraine; ^6^CFisUC, Department of Physics, University of Coimbra, CoimbraPortugal

**Keywords:** candidate division JS1, iron reduction, methanogens, ANME, subsurface sediments, North Sea, SMT, anaerobic oxidation of methane

## Abstract

Iron reduction in subseafloor sulfate-depleted and methane-rich marine sediments is currently a subject of interest in subsurface geomicrobiology. While iron reduction and microorganisms involved have been well studied in marine surface sediments, little is known about microorganisms responsible for iron reduction in deep methanic sediments. Here, we used quantitative PCR-based 16S rRNA gene copy numbers and pyrosequencing-based relative abundances of bacteria and archaea to investigate covariance between distinct microbial populations and specific geochemical profiles in the top 5 m of sediment cores from the Helgoland mud area, North Sea. We found that gene copy numbers of bacteria and archaea were specifically higher around the peak of dissolved iron in the methanic zone (250–350 cm). The higher copy numbers at these depths were also reflected by the relative sequence abundances of members of the candidate division JS1, methanogenic and *Methanohalobium*/ANME-3 related archaea. The distribution of these populations was strongly correlated to the profile of pore-water Fe^2+^ while that of *Desulfobacteraceae* corresponded to the pore-water sulfate profile. Furthermore, specific JS1 populations also strongly co-varied with the distribution of *Methanosaetaceae* in the methanic zone. Our data suggest that the interplay among JS1 bacteria, methanogenic archaea and *Methanohalobium*/ANME-3-related archaea may be important for iron reduction and methane cycling in deep methanic sediments of the Helgoland mud area and perhaps in other methane-rich depositional environments.

## Introduction

The involvement of microorganisms in electron-accepting processes (EAP) in marine sediments results in the formation of redox zones which may overlap with one another (Canfield and Thamdrup, 2009). Regardless, specific EAP may still dominate in certain zones with the result that microorganisms that are involved in these distinct processes are more dominant (Jorgensen et al., 2012). For example, at the sulfate-methane transition (SMT) of marine sediments sulfate reducers belonging to the *Desulfococcus/Desulfosarcina* (DSS) group and methanotrophic archaea (ANME-1, 2, and 3) are often found in relatively higher proportion (Lloyd et al., 2006; Harrison et al., 2009; Knittel and Boetius, 2009) compared to deeper methanic zones. Due to overlapping redox zonation in marine sediments, it may be challenging to accurately correlate down-core microbial community distribution to geochemical profiles. However, Jorgensen et al. (2012) have recently shown that the depth-wise distribution of archaeal populations, Marine Group I, are linked to nitrate profiles of deep sediments from the arctic mid-ocean ridge, thus buttressing their suspected role in the nitrogen cycle (Durbin and Teske, 2011).

Iron reduction is a major electron-accepting process in marine surface sediments and organisms known to be involved in this process have been studied (Roden and Lovley, 1993; Vandieken et al., 2006b; Nickel et al., 2008; Vandieken and Thamdrup, 2013). While members of the *Geobacteraceae* are considered as the major populations mediating metal reduction in freshwater sediments (Coates et al., 1996), other members of the *Desulfuromonadales* (e.g., *Desulfuromusa* and *Desulfuromonas*) are considered to be important in marine sediments (Roden and Lovley, 1993; Vandieken et al., 2006a). Based on most-probable-number (MPN) cell counts, members of the *Desulfuromonadales* were the most abundant iron-reducing bacteria (65% of the total bacteria population) in surface sediments from Aarhus Bay (Vandieken and Thamdrup, 2013).

In contrast to marine surface sediments, microbes involved in iron reduction in subsurface sediments have not been identified so far. At a number of sites elevated amounts of dissolved iron (i.e., Fe^2+^) in pore-water have been observed: for example, in sediments of the Amazon Fan (Flood et al., 1995; Kasten et al., 1998), Peru Margin (D’Hondt et al., 2004), Sea of Okhotsk (Wallmann et al., 2008), Argentine Basin (Hensen et al., 2003; Riedinger et al., 2005, 2014), Zambesi Fan (März et al., 2008), Aarhus Bay (Holmkvist et al., 2011) and Bothnian Sea (Slomp et al., 2013; Egger et al., 2014). To explain the source of dissolved iron in subsurface sediments, a number of hypotheses may be considered. In Aarhus Bay sediments, a chemical reaction between buried iron(III) minerals and hydrogen sulfide diffusing downward from the SMT has been suggested to explain the formation of Fe^2+^ (Holmkvist et al., 2011). In the sulfate-depleted methanic zone of sediments of the Argentine Basin, it is argued that iron reduction is most likely coupled to the anaerobic oxidation of methane (Fe-AOM; Riedinger et al., 2014). This process has been suggested to be directly or indirectly linked to microbial activity (Beal et al., 2009). In addition, oxidation of products of organic matter fermentation such as acetate and hydrogen coupled to iron reduction by dissimilatory iron-reducing microorganisms (Roden and Lovley, 1993) may also be a possibility. Lastly, the non-dissimilatory reduction could be a potential pathway in which iron(III) oxides serve as an electron sink during fermentation of complex organic matter (Lovley and Phillips, 1986; Dobbin et al., 1999).

Elevated concentrations of dissolved iron measured in the pore-water of sediments below the SMT (methanic zone) in the Helgoland mud area have prompted us to investigate the potential involvement of certain microbial populations in the reduction of iron therein. In this study, we use molecular ecology techniques such as quantitative PCR and 454-pyrosequencing as well as geochemical measurements to estimate cell numbers and determine the proportion of distinct bacteria and archaea populations in the sediments of the Helgoland mud area down to over 530 cm below sea floor in relation to the dissolved iron profile.

## Materials and Methods

### Site and Sampling Description

**FIGURE 1:**
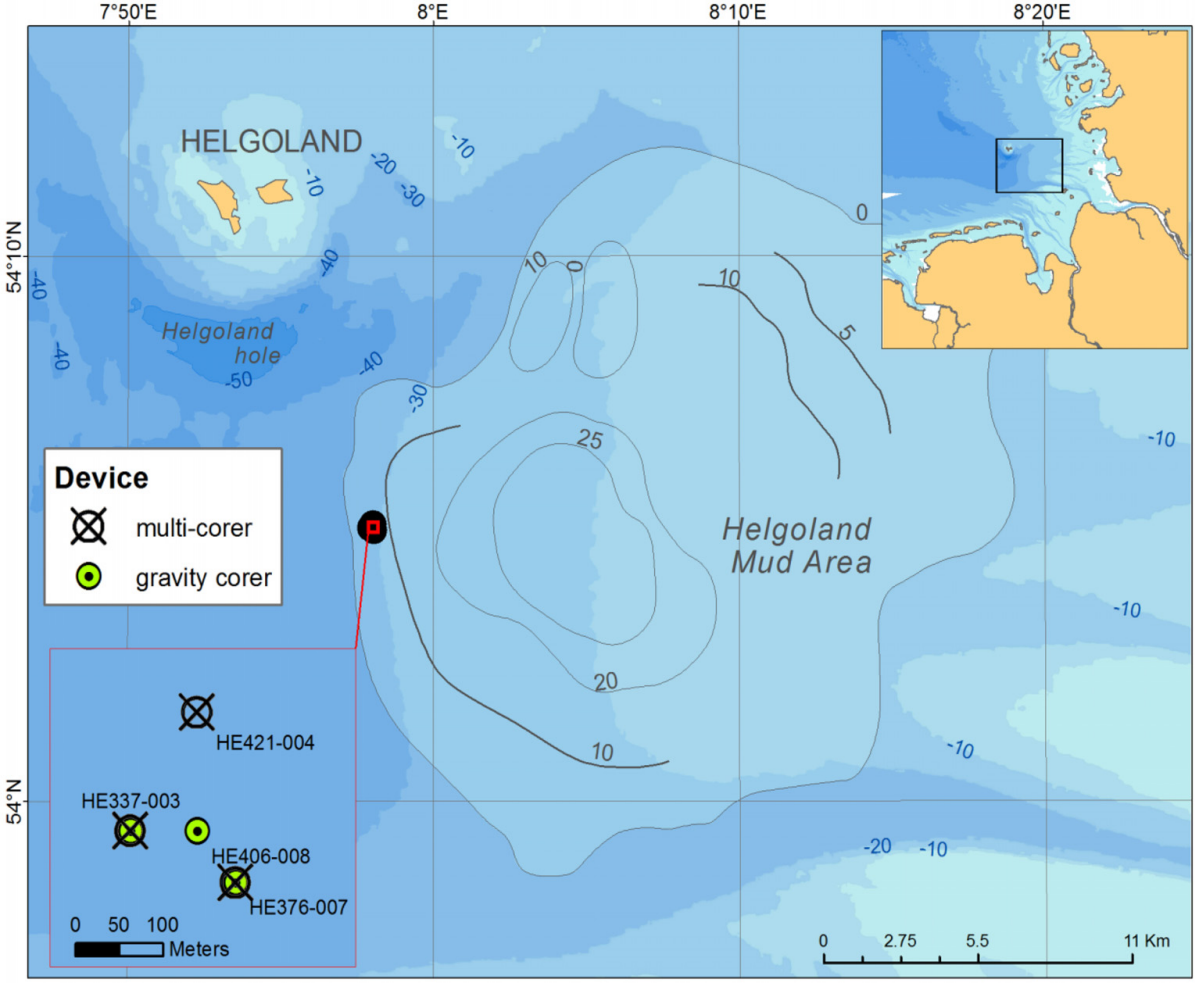
**Sampling sites in the Helgoland mud area**.

The Helgoland mud area (**Figure 1**) in the German Bight of the North Sea extends over ∼500 km^2^ and has a water depth of less than 30 m (Hebbeln et al., 2003). It represents one of the few depocenters of fine-grained sediments in the North Sea. The average sedimentation rate was estimated to have been high (13 mm yr^-1^) between 750–1550 before present. Presently, it is at 1.6 mm yr^-1^ (Hebbeln et al., 2003). During RV HEINCKE cruise HE376 in April 2012, subsurface sediment samples were collected from site HE 376-007 (**Table 1**) using a gravity corer (GC; 5 m core length). The upper 20–30 cm of sediment is generally lost when using a GC. Therefore, a multi corer (MUC) was used to collect undisturbed surface sediments (30 cm core length) from the same site during cruises with RV UTHÖRN in September 2012 (UT-2012; **Table 1**) and with RV HEINCKE in April 2014 (HE421-004; **Table 1**). The 5 m-long gravity core HE376-007-5 was cut in 25 cm sections, and subsamples of each section, taken with sterile 10 ml cut-off syringes, were frozen at -80°C for molecular analyses. For the MUC core UT-2012, only the top 10 cm of the 30 cm-long sediment core was processed for molecular analysis as this corresponded to the depth at which high dissolved iron was measured based on previous geochemical investigations at this study site. The sediment samples from the top 10 cm were homogenized and stored in separate 50 ml Falcon tubes at -80°C until further use. For pore-water and solid-phase analyses, a parallel gravity core at site HE376-007 was taken and sampled. Pore-water was retrieved every 25 cm by means of rhizon samplers which have an average pore size of 0.15 μm according to procedures described by Seeberg-Elverfeldt et al. (2005) and Dickens et al. (2007). For the measurement of methane concentrations, 5 ml of wet sediment were taken with cut-off syringes and were inserted into 50 ml headspace vials pre-filled with 20 ml of saturated NaCl solution. The vials were tightly closed with rubber septa, sealed with aluminum crimps and stored inverted at +4°C to minimize methane loss.

**Table 1:** Sampling information of sediment samples analyzed in the study.

	Gear	Date	Site	Coordinates
Pore-water profile	Gravity corer	April (2012)	HE376-007	54°5.00′N7°58.05′E
	Multi corer	April (2014)	HE421-004	54°5.10′N7°58.01′E
Molecular analysis	Multi corer	September (2012)	UT-2012	54°5.00′N7°58.05′E
	Gravity corer	April (2012)	HE376-007	54°5.00′N7°58.05′E
Sequential sediment extractions	Gravity corer	April (2012)	HE376-007	54°5.00′N7°58.05′E
Mössbauer spectroscopy	Gravity corer Multi corer	July (2013), April (2014)	HE406-008, HE421-004	54°5.01′N7°58.01′E 54°5.10′N7°58.01′E

For Mössbauer spectroscopy, GC, and MUC cores collected during RV HEINCKE cruises HE406 and HE421 conducted in July 2013 and April 2014, respectively, were used (**Table 1**). In the home lab, the GC retrieved from site HE406-008 was cut into 25 cm sections and the top 10 cm of the MUC core (UT-2012) were sampled as described above. 10 g of wet sediment were transferred into wide-mouth glass vials under a stream of argon gas (99.998% purity, Linde, Germany), frozen at -20°C and then freeze-dried to avoid air oxygen contamination. The freeze-drier chamber was filled with argon, lyophilized samples were removed immediately, sealed with rubber septa, and headspaces were flushed with argon on a manifold.

### Geochemical Analyses

#### Pore-Water Measurements

For the analysis of dissolved iron, 1 ml pore-water aliquots were transferred into cuvettes pre-filled with 50 μl of Ferrospectral solution immediately after pore-water retrieval on board the RV HEINCKE. Dissolved iron concentrations were measured photometrically at a wavelength of 565 nm. 1.5 ml subsamples of pore-water were added to a 2.5% zinc acetate solution on board in order to fix all sulfide present as ZnS. After the cruise, sulfide concentrations were analyzed in the laboratories of the AWI using a spectrophotometer applying the methylene blue method (Cline, 1969). After dilution of pore-water samples, concentrations of ammonium were measured as described by Hall and Aller (1992). Pore-water sulfate concentrations were determined in pore-water aliquots (1:50 dilution) using a Metrohm Compact IC 761 ion chromatograph.

Determination of methane concentrations in pore-water was carried out in the laboratory of the AWI, Bremerhaven. This was done by injecting 20–100 μl of the headspace gas from closed vials containing wet sediments into a Thermo Finnigan TRACE GC equipped with a packed column and an integrated flame ionization detector (FID).

#### Sequential Extraction of Iron Minerals

Iron adsorbed to particle surfaces as well as bound in carbonates and iron (oxyhydr)oxides was extracted from the sediment using a sequential extraction procedure developed by Poulton and Canfield (2005). In brief, about 80 mg of dry sediment from the gravity core from site HE 376-007 (**Table 1**) were exposed to a sequence of four leaching agents and shaken for a defined period of time at room temperature (**Table 2**). Iron concentrations in the extracts were determined using an ICP-OES (Iris Intrepid, Thermo Elemental) with a relative standard deviation of less than 5%. In order to circumvent matrix effects, we prepared the standards for the ICP-OES with the same relative amount (1:20) of leaching agent as is in the extracts.

**Table 2:** Sequential extraction procedure of iron minerals in sediment samples.

Step	Abbreviation	Extraction Agent	Target Fractions
1	FeCarb	1 M Na-acetate (pH 4.5) for 24 h	Adsorbed Fe, Fe carbonates
2	FeOX1	1 M hydroxylamine-HCl in 25 %v/v acetic acid (pH 2) for 24 h	Amorphous or poorly crystalline Fe (oxyhydr)oxides, mainly ferrihydrite and lepidocrocite
3	FeOX2	0.35 M acetic acid/0.2 M Na-citrate/0.28 M Na-dithionite (pH 4.8) for 2 h	Crystalline Fe (oxyhydr)oxides, mainly goethite, hematite
4	FeMag	0.2 M ammonium oxalate/0.17 M (pH 3.2) oxalic acid for 6 h	Crystalline Fe (oxyhydr)oxides, mainly (titano)magnetite, maghemite

#### Mössbauer Spectroscopy

^57^Fe-Mössbauer spectra were recorded in transmission geometry with an 8 mCi ^57^Co source embedded in a rhodium matrix using a conventional constant-acceleration Mössbauer spectrometer (WissEL GmbH, Starnberg, Germany) equipped with a bath helium cryostat. The absorbers were prepared by placing the powdered samples of about 200 mg between acryl platelets of the sealed sample holder. Isomer shifts are given relatively to iron metal at ambient temperature. Simulations of the experimental data were performed with the *Recoil* program (Lagarec and Rancourt, 1997).

### Molecular Ecology Analyses

#### Nucleic Acid Extraction

DNA was extracted from 0.5 to 0.6 g of wet sediment following Lueders et al. (2004) with modifications. Cells were disrupted twice for 45 s by bead-beating. DNA was precipitated with 0.2 volumes of 7.5 M ammonium acetate and one volume of isopropanol for 1 h at room temperature and collected by centrifugation at 17,950 *g*, at 4°C for 20 min. The final DNA pellet was dissolved in 50 μl elution buffer (Qiagen, Hilden, Germany). DNA concentration was measured using NanoDrop 1000 spectrophotometer (Peqlab Biotechnologie, Erlangen, Germany). DNA extracts from all samples were stored at -20°C until further processing.

#### Quantitative PCR (Q-PCR)

To estimate cell abundances through copy numbers of bacterial and archaeal 16S rRNA genes, Q-PCR was performed using DNA extracts from *Escherichia coli* strain SB1 and *Methanosarcina barkeri* (DSM 800) as standards, respectively. Standard templates were prepared by amplifying the 16S rRNA genes using primer pairs 27F and 907R (Lane, 1991) for bacteria and Ar109F (Grosskopf et al., 1998), and Ar912r (Lueders and Friedrich, 2000) for archaea. The concentrations of purified PCR products were determined using Qubit 2.0 fluorometer (Invitrogen, Darmstadt, Germany). Standard curves were prepared using standard templates. Primer pair 338F (Muyzer et al., 1993) and 518R (Muyzer et al., 1993) were used for quantifying bacterial 16S rRNA gene copies and primer pair Ar806F (Takai and Horikoshi, 2000) and Ar912rt (Lueders and Friedrich, 2002) for archaeal gene copies. Each Q-PCR reaction contained a total volume of 20 μl: 10 μl of master mix (MESA GREEN qPCR master mix, Eurogentec, Cologne, Germany), 0.5 μl of each primer (0.25 μM each; final concentration), 5 μl of RNase-free water, and 4 μl of template DNA. Amplification efficiency of not less than 90% and slope of -3.6 was obtained. Gene copy numbers per gram of wet sediment were calculated using the formula: gene copies = (quantity of DNA [g/μl]/size of amplicon (bp)) × (Avogadro’s constant/660) assuming that the average weight of 1 bp is equal to 660 daltons.

#### Pyrosequencing and Sequence Analysis

DNA samples from depths of 0–5 cm and 5–10 cm (surface sediments), 30–55 cm (SMT), 180–205 cm, 230–255 cm, 305–330 cm, 355–380 cm, and 480–505 cm (methanic zones) were selected for 454 FLX pyrosequencing at Molecular and Research Testing Laboratory, (Lubbock, TX, USA). Primer pairs used for sequencing bacterial 16S rRNA genes were 104F (5′-GGC GVA CGG GTG AGT AA-3′) and 530R (5′-CCG CNG CNG CTG GCA C-3′; Wang and Qian, 2009). Primers 349F (5′-GYG CAS CAG KCG MGA AW-3′) and 806R (5′-GGA CTA CVS GGG TAT CTA AT-3′; Takai and Horikoshi, 2000) were used for archaea. Sequence raw data (SFF files) were subjected to downstream processing using QIIME version 1.6 (Caporaso et al., 2010). Barcodes and low quality sequences (less than 200 bp) were removed using Amplicon Noise (Quince et al., 2011). Taxonomic classification was done based on the Greengenes data base v12_10. The number of sequences per sample obtained can be found in **Table 3**. Raw sequences obtained from pyrosequencing analyses have been uploaded to the MG-RAST metagenomics analysis server for public access (Meyer et al., 2008; MG-RAST ids: 4612914.3, 4612912.3, 4612913.3, 4612915.3, 4612916.3, 4612917.3, 4612911.3, 4612918.3, 4612906.3, 4612904.3, 4612905.3, 4612907.3, 4612908.3, 4612909.3, 4612903.3, 4612910.3).

**Table 3:** Number of pyrosequencing-generated bacterial and archaeal 16S rRNA gene sequences analyzed per depth sampled.

Sediment depth	Bacteria	Archaea
0–5 cm	2018	*
5–10 cm	1753	*
30–55 cm	23683	3185
180–205 cm	21403	1579
230–255 cm	14391	16984
305–330 cm	17018	16984
355–380 cm	25792	15079
480–505 cm	21388	2227

*Not applicable.

For phylogenetic analysis, sequences were aligned using ARB version 6.0.2 (Ludwig et al., 2004) and the closest neighbor and type strain sequences were identified and extracted using the SILVA non-redundant reference database [SSU Ref NR 99, Version 119 (Quast et al., 2013)]. Tree topologies were calculated with the maximum likelihood and neighbor joining algorithms as implemented in MEGA 6 (Tamura et al., 2013).

### Statistical Analysis

In order to test for association/correlation between depth-wise distribution of microbial populations and specific geochemical profiles, spearman correlation (ρ) was used. This calculation was done using R software (http://www.r-project.org).

## Results

### Geochemical Profiles

**FIGURE 2:**
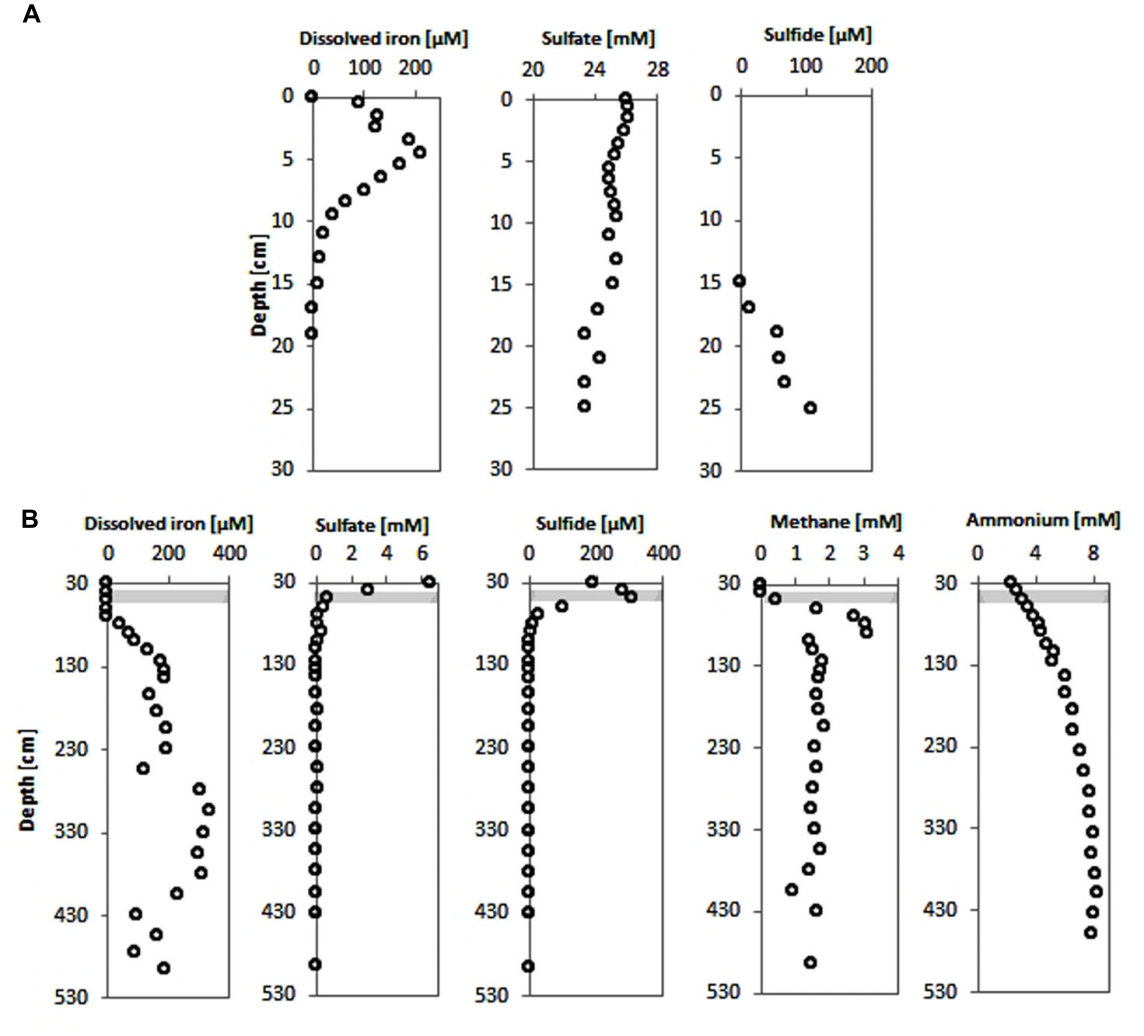
**Pore-water concentration profiles of geochemical parameters of surface and deep sediments of the Helgoland mud area. (A)** Pore-water profiles of dissolved iron, sulfate and sulfide in surface sediments (HE421-004). **(B)** Pore-water profiles of dissolved iron, sulfate, sulfide, methane, and ammonium in deeper sediments (HE376-007-2). Loss of the upper 20–30 cm during gravity corer sampling is taken into consideration by defining the starting depths as 30 cm in geochemical profiles from deep sediment cores. Gray bar represents the SMT.

Pore-water sulfate concentrations showed a linear decrease with depth from ∼6 mM at the top of the gravity core HE376-007-2 down to below detection limit at a depth of about 70 cm (**Figure 2B**). Concomitantly, methane concentrations increased below this depth reaching a maximum concentration of 3.2 mM around 105 cm (**Figure 2B**). Below 105 cm, methane concentrations ranged between 1 and 2 mM. Sulfide concentrations increased downward up to 350 μM at a depth of about 50 cm and were completely depleted in the methanic zone (**Figure 2B**). Pore-water ammonium gradually increased down-core from 2 mM at the top of the gravity core to more than 8 mM in the methanic zone (**Figure 2B**).

Dissolved iron was detected in the top 15 cm of sediments of the MUC core HE421-004 with a maximum of 210 μM found at 4.5 cm sediment depth (**Figure 2A**). Below the Fe^2+^-containing zone hydrogen sulfide increased downward to 108 μM at 25 cm depth. Sulfate showed only slightly depleted values compared to bottom water concentrations (**Figure 2A**).

In the upper part of the gravity core, dissolved iron could not be detected (**Figure 2B**). Measurements of pore-water samples from the methanic zone showed a gradual increase in dissolved iron as sulfate was depleted (**Figure 2B**). Dissolved iron in the pore-water reached highest concentrations (∼330 μM) in the depth interval 280–380 cm (**Figure 2B**).

### Sequential Iron Mineral Extraction

**FIGURE 3:**
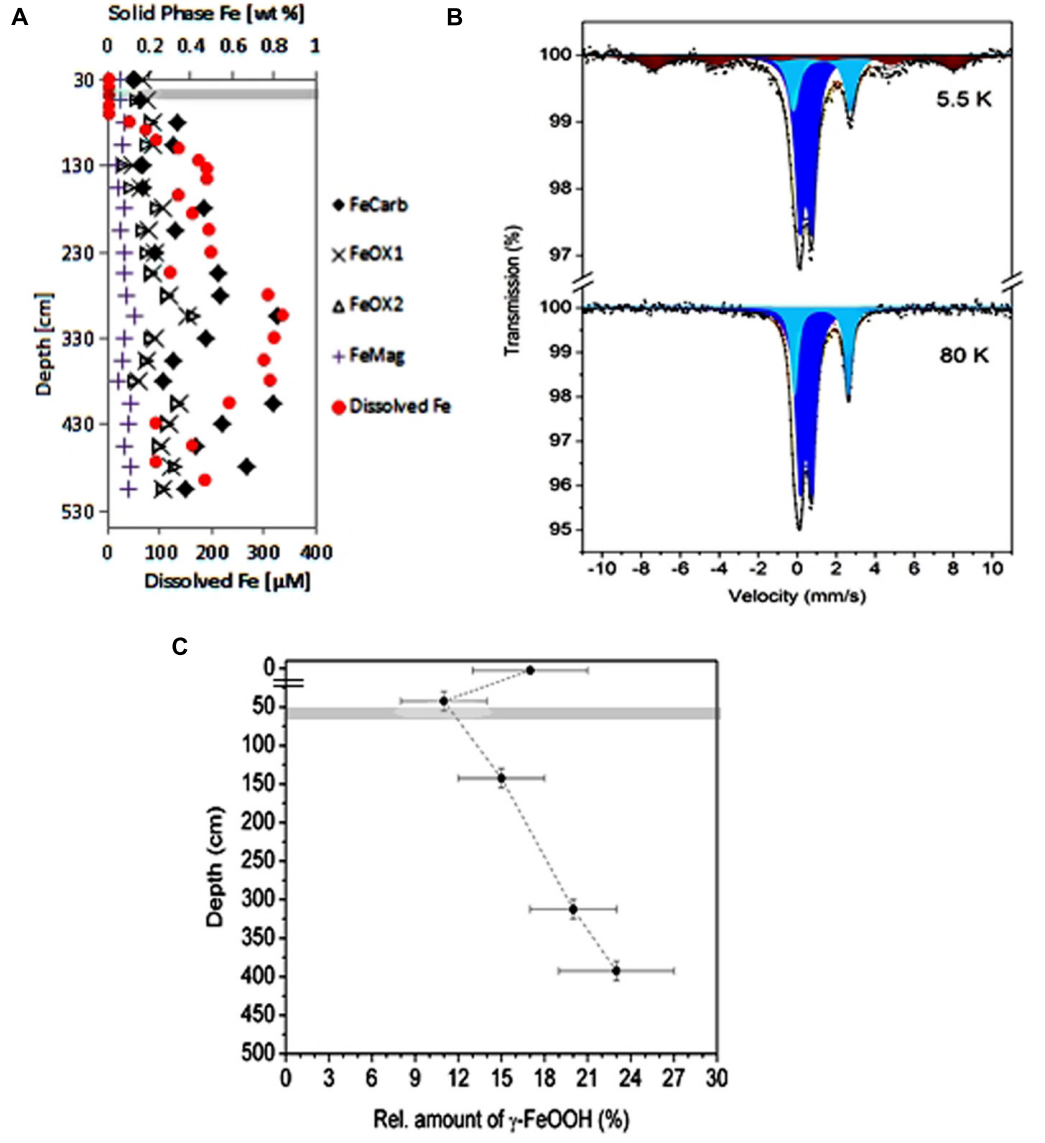
**Iron speciation in the Helgoland mud area sediment. (A)** Sequential extraction of iron minerals in sediment core samples (HE376-007-5). Filled circles in red color, pore-water profile of dissolved iron at the same site for comparison with the distributions of solid phase iron minerals. **(B,C)** Show results of Mössbauer analysis of sediments samples from the surface and deep sediments (HE 421-004 and HE406-008). **(B)** Spectra of iron minerals detected in a sediment sample taken from 380 to 405 cm at 5.5 K (upper panel) and 80 K (lower panel). Illite, light and dark blue; lepidocrocite, brown. **(C)** Depth-wise distribution of the amount of lepidocrocite. The break in the depth axis is the demarcation between samples from surface and deep sediments. Gray bar denotes the SMT.

Sequential extraction of iron minerals yielded four operationally defined phases as shown in **Table 2** and **Figure 3A**. The most abundant phases are those designated FeCarb_,_ containing adsorbed iron and iron carbonates. These fractions varied between 0.17 and 0.82 weight percent down the depth of the sediment core. FeOX1 and FeOX2 fractions containing amorphous (mainly ferrihydrite and lepidocrocite) and crystalline (mainly goethite and hematite) Fe(oxyhydr)oxides, respectively showed similar concentrations at all depths sampled. These fractions both ranged between 0.14 and 0.41 weight percent. The FeMag fraction assumed to contain mostly magnetite was least abundant and varied between 0.04 and 0.13 weight percent. In general, oxidized iron minerals were found to be most abundant around 305 cm, which corresponded to the depth with the highest dissolved iron concentrations measured.

### Mössbauer Spectroscopy

In order to determine the exact nature of the reactive iron mineral(s) present in the Helgoland mud area, Mössbauer spectra of selected sediment samples [0–5 cm (MUC; HE421-004), 30–55 cm, 130–155 cm, 305–330 cm, and 380–405 cm (GC; HE406-008)] were recorded at 5.5 K, 80 K, and 293 K. The spectrum of the sediment from the 380–405 cm depth at 5.5 K is shown in **Figure 3B**. The detailed analysis of the Mössbauer data revealed the presence of illite and lepidocrocite. Thus, two quadrupole doublets with δ = 0.43(1) mm/s, ΔE_Q_ = 0.62(2) mm/s and δ = 1.25(1) mm/s, ΔE_Q_ = 2.93(2) mm/s correspond to high-spin Fe(III) and high-spin Fe(II) of illite respectively, which is in accordance with those reported elsewhere (Murad and Wagner, 1994). The magnetic sextet fitted with δ = 0.34(1) mm/s, H_hf_ = 467(6) mm/s corresponds to Fe(III) sites of lepidocrocite. The absence of magnetic sites at 80 K (**Figure 3B**) and 293 K (data not shown) spectra excludes aside from lepidocrocite, the presence of other magnetic iron oxides or hydroxides (Greenwood and Gibb, 1971) abundant enough to be detected by our system (detection limit: 3% of total Fe). Although the relation between Fe(III) and Fe(II) doublets of illite does not show significant dependence on depth (data not shown), the relative amount of the lepidocrocite does vary with depth (**Figure 3C**).

### 16S rRNA Gene Copy Numbers

**FIGURE 4:**
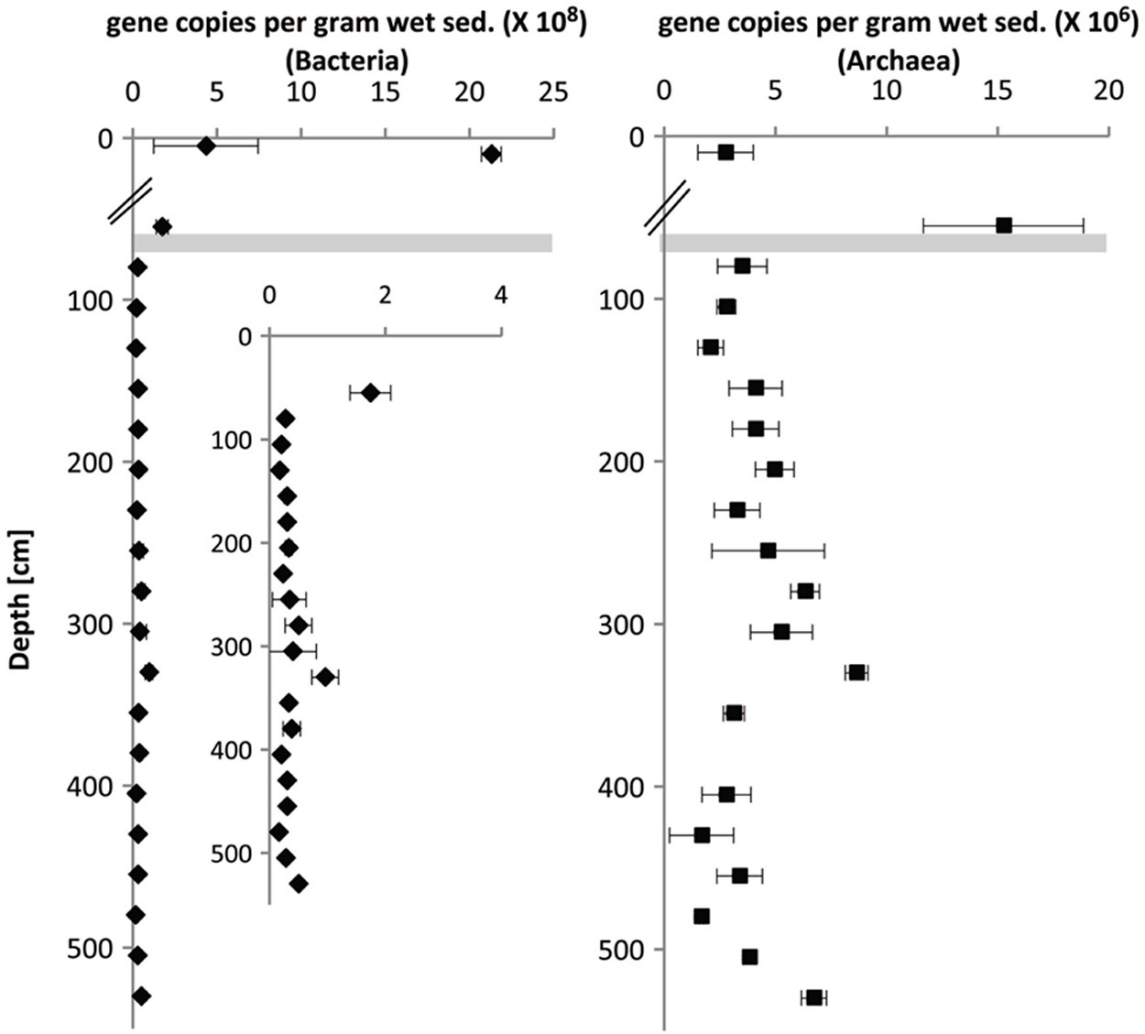
**Depth-wise estimation of bacterial and archaeal 16S rRNA gene copies based on Q-PCR**. The breaks in the *x*-axes are demarcations between surface (top 10 cm, UT-2012) and deep sediments (≥30 cm, HE376-007-5). Error bars represent the SD of three technical replicates (*n* = 3). Gray bar denotes the SMT.

Gene copy numbers in surface sediments, SMT and methanic zone were estimated by quantifying bacterial and archaeal 16S rRNA genes using qPCR (**Figure 4**). In general, bacterial 16S rRNA gene copies were highest in surface sediments (10^9^ copies/grams wet sediment), while gene copies of archaea were highest in the SMT (10^8^ copies/gram wet sediment). Archaeal gene copies were one order of magnitude lower than bacterial gene copies both in the SMT (10^7^ vs. 10^8^ respectively) and at each depth of the methanic zone sampled (10^6^ vs. 10^7^ respectively). In surface sediments, bacterial gene copies dominated archaeal gene copies by three orders of magnitude (10^9^ vs. 10^6^ copies/gram wet sediment). Interestingly, in the methanic zone, both archaeal and bacterial gene copies were highest at 275–350 cm corresponding to the depths of maximum pore-water iron concentrations.

### Known Potential Iron Reducers in Surface Sediments

**FIGURE 5:**
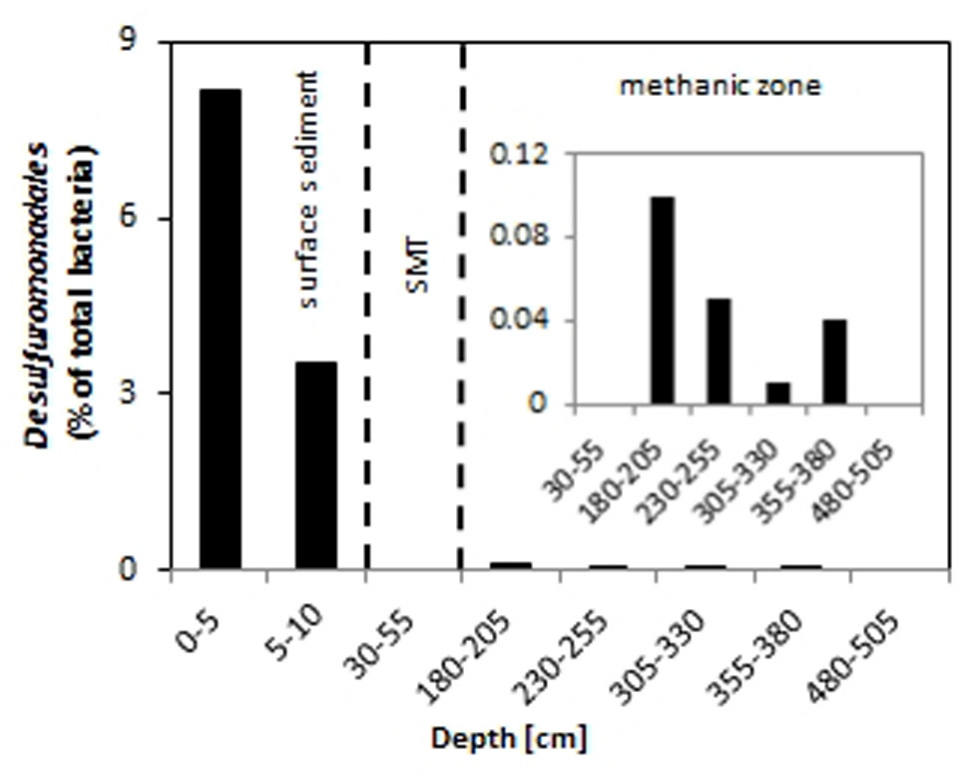
**Relative abundance of *Desulfuromonadales* in the surface (top 10 cm, core UT-2012), and deep sediments (≥30 cm, core HE376-007-5)**. The inset shows a magnified representation of the values from 30 to 505 cm. SMT, 30–55 cm.

Among the sequences retrieved were those from bacteria that are known to possess iron-reducing capabilities such as members of the *Desulfuromonadales*. Relative abundances of *Desulfuromonadales* were 8.2 and 3.5% of the total bacterial community at 0–5 cm and 5–10 cm, respectively (**Figure 5**). *Desulfuromonadales* were not detected in the SMT (30–55 cm). However, they could be detected at all depths of the methanic zone sampled except at 480–505 cm. In the methanic zone, relative abundances of *Desulfuromonadales* at the depths sampled range from 0.01 to 0.1%.

### Relationship of Distinct Microbial Populations to the Deep Pore-Water Iron Profile

**FIGURE 6:**
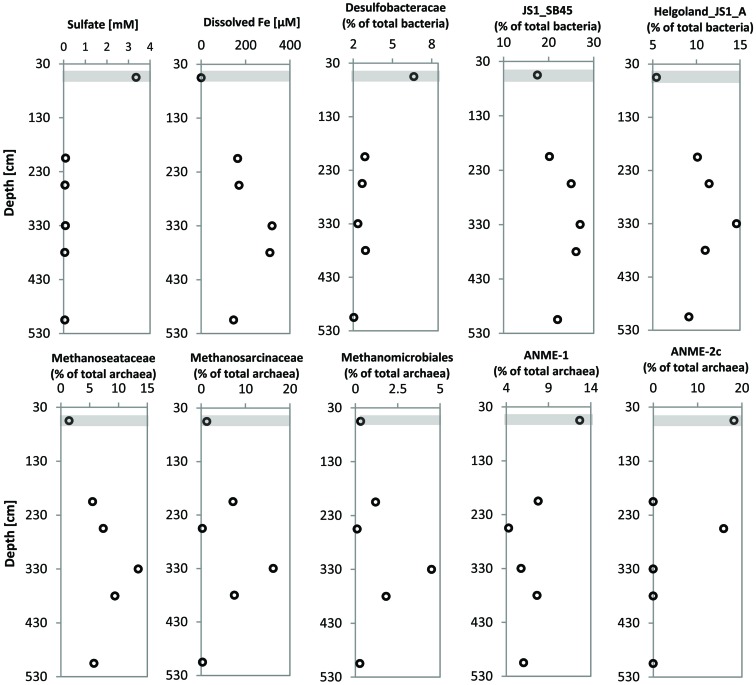
**Profiles showing the distribution of specific microbial populations from gravity core HE376-007-5 at selected depths and corresponding concentrations of pore-water sulfate and dissolved iron at the SMT and in the methanic zone (HE376-007-2)**. Concentrations plotted for pore-water profiles of sulfate and dissolved iron here are mean concentrations of three depth interval of 30 cm each, for which molecular analyses were performed (**Table 3**). Gray bar denotes the SMT.

**FIGURE 7:**
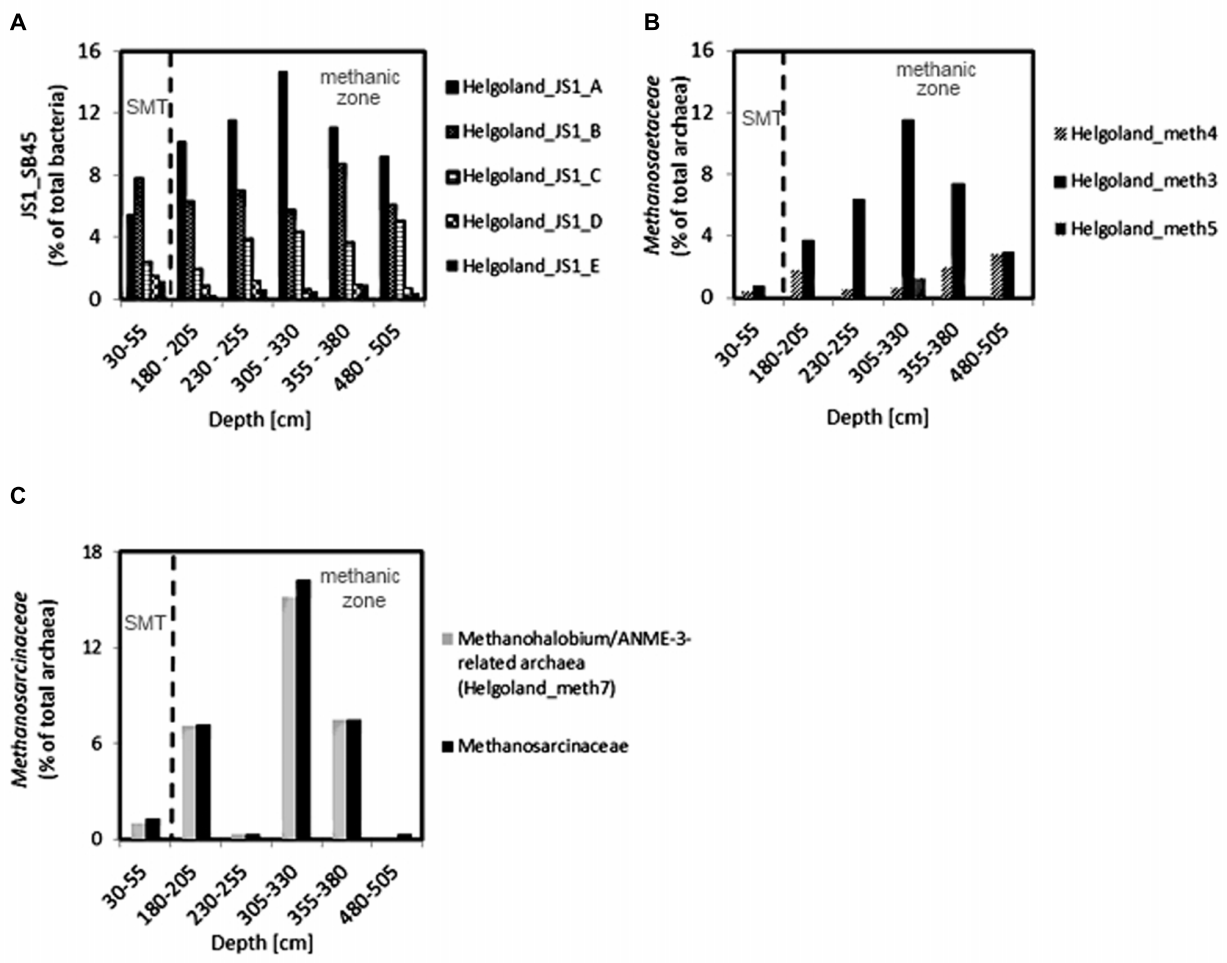
Depth-wise distribution of selected JS1 bacteria, and archaeal OTUs. **(A)** Relative abundances of most abundant JS1 bacteria OTUs. **(B)** Relative abundance of *Methanosaeta*-related OTUs. **(C)** Distribution of *Methanohalobium*/ANME-3-related archaea (Helgoland_meth7) as the dominant members of *Methanosarcinaceae*. Helgoland_JS1_A, Helgoland_meth3, and Helgoland_meth7 follow the similar depth-wise distribution patterns and show highest abundance at 305–330 cm (dissolved Fe peak).

**FIGURE 8:**
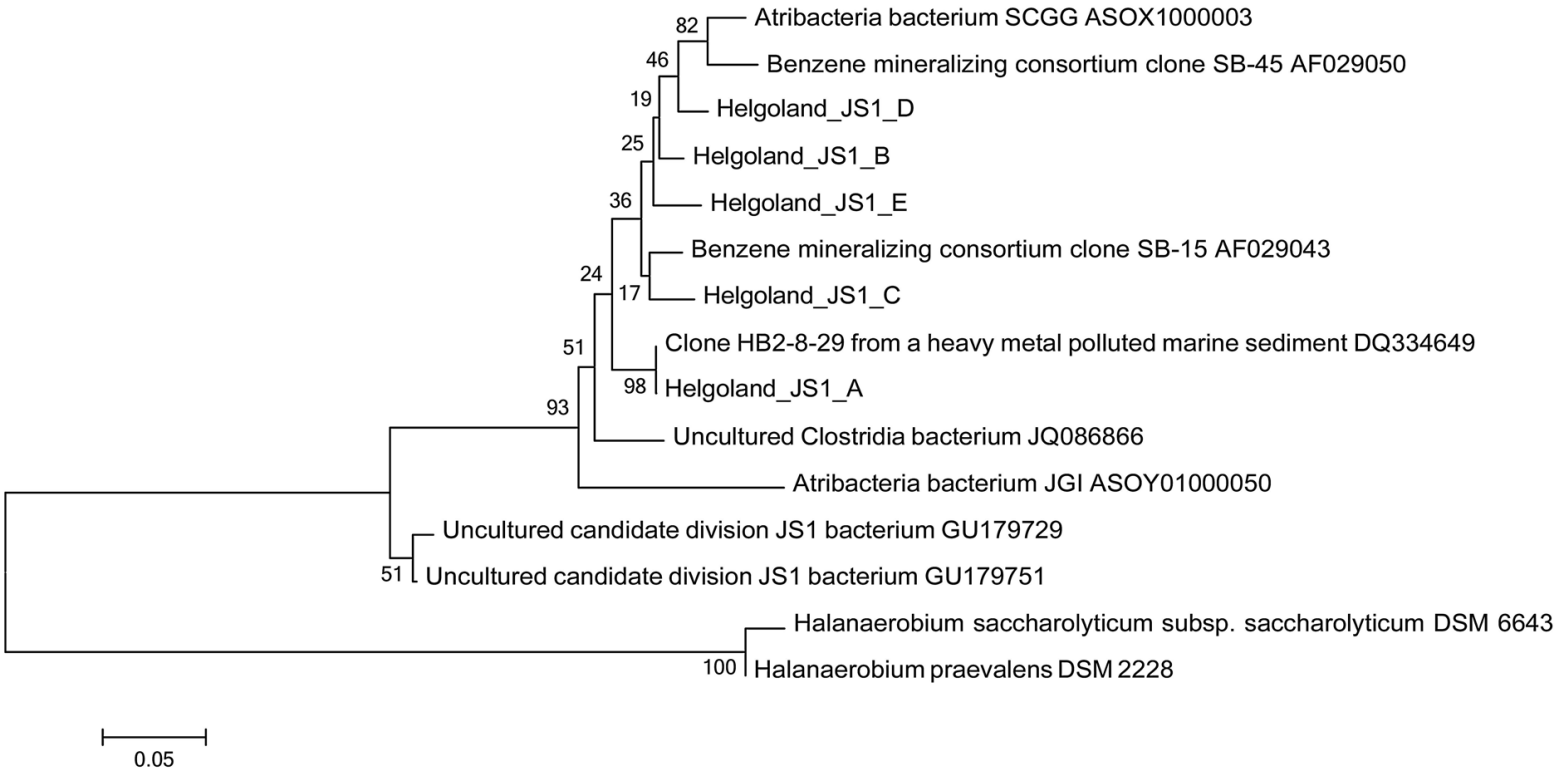
Neighbor-joining tree of 16S rRNA gene sequences showing the phylogenetic affiliation of the most abundant JS1 bacteria sequences (Helgoland_JS1_A-E). Helgoland_JS1_A OTU is closely related to clone from a heavy metal-contaminated site (DQ334649). Scale bar represents 5 base substitutions per 100 nucleotides. *Halanaerobium saccharolyticum* and *H. praevalens* were used as outgroup references.

**FIGURE 9:**
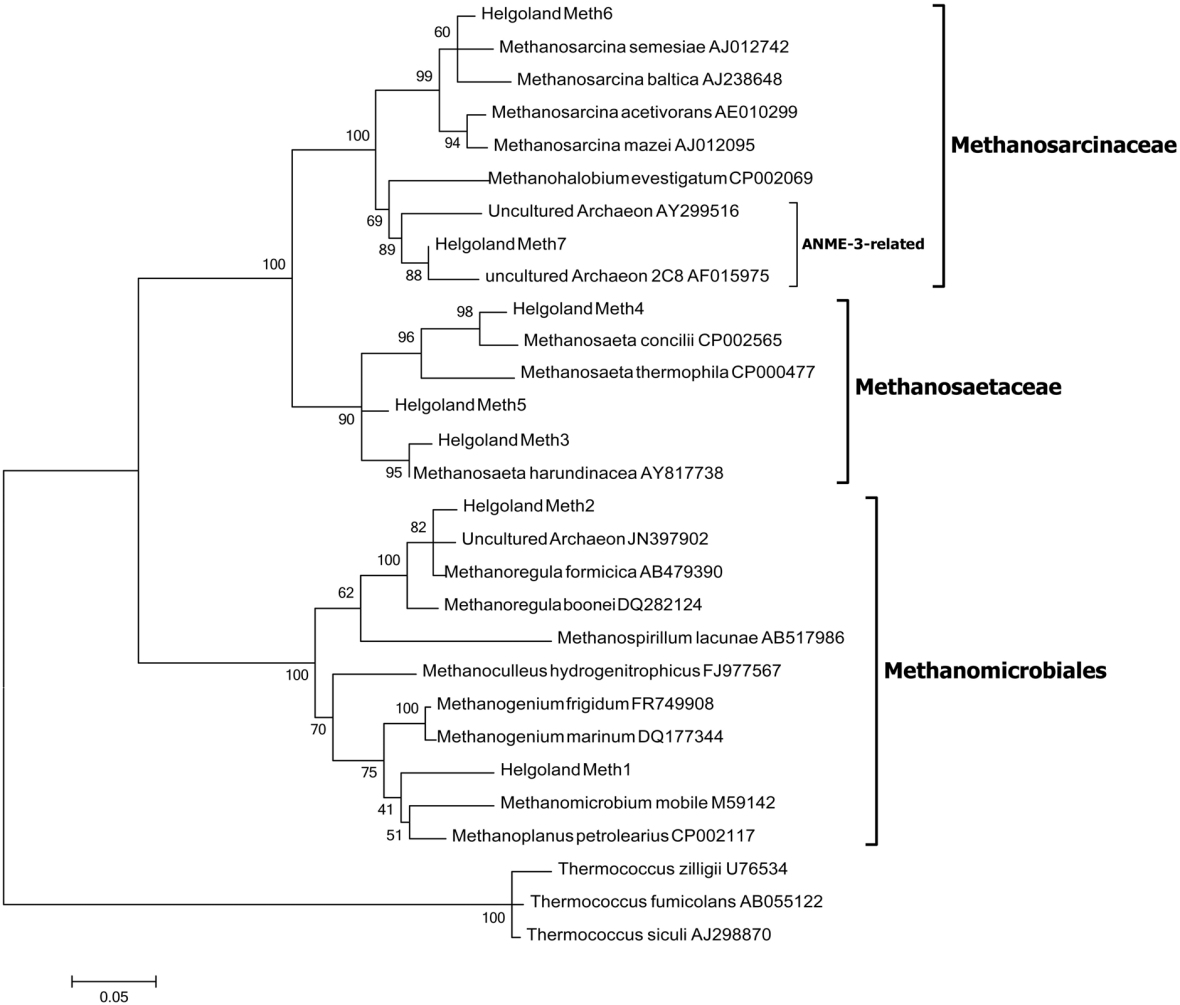
Maximum likelihood tree of 16S rRNA gene sequences showing the phylogenetic affiliations of the methanogen-related sequences detected in the Helgoland mud area. Scale bar represents 5 base substitutions per 100 nucleotides. *Thermococcus zilligii*, *T. fumicolans,* and *T. siculi* served as outgroup references.

Since members of the *Desulfobacteraceae* live in syntrophic associations with ANME populations in the reduction of sulfate coupled to the oxidation of methane (Boetius et al., 2000; Knittel and Boetius, 2009), they should be more abundant in the SMT with higher sulfate concentrations compared to the methanic zone where sulfate is almost completely depleted (**Figure 2B**). As predicted, we found that the distribution of the *Desulfobacteraceae* did correspond to the sulfate pore-water profile (**Figure 6**). We then checked for populations that corresponded to the dissolved iron profile and found that the depth-wise distribution of the JS1_SB45 candidate division showed a strong positive covariance (ρ = 0.943; *p*-value = 0.004; **Table 4**). Further probing of the first five most abundant operational taxonomic units [OTUs; Helgoland_JS1_A to JS1_D (**Figure 7A**)] in the SB45 lineage at each depth sampled revealed that an OTU (Helgoland_JS1_A, **Figure 8**), closely related (100% sequence identity; ∼200 bp sequence length) to clone HB2-8-29 (DQ334649), was the main determinant of the positive relationship between the SB45 lineage and the dissolved iron profile (ρ = 0.943; *p*-value = 0.004). Helgoland_JS1_A was also found to mirror the distribution of members of the *Methanosaetaceae* and more specifically a clone 100% identical in sequence to the *Methanosaeta*-related clone MNO686arcE10 (GU996834; ρ = 1.000; *p*-value < 0.005). This *Methanosaeta*-related OTU is designated here as Helgoland_Meth3 (Figures **Figure 7B** and **Figure 9**). In addition, other sequences related to methanogenic archaea, *Methanosarcinaceae* and *Methanomicrobiales,* generally showed a similar trend to *Methanosaetaceae* and Helgoland_JS1_A (**Figure 6**). However, at 230–255 cm, they were detected in very low abundances (0.3 and 0.1% respectively). Instead, the relative abundance of ANME-2c archaea sequences increased at the same depth (**Figure 6**) to ∼16% of total archaea. Both ANME-1 and ANME-2c did not show covariance with neither of Helgoland_JS1_A, *Methanosaetaceae* nor the dissolved iron profile (**Figure 6**). In the depths sampled, Helgoland_Meth7 (**Figure 8**), closely related to uncultured archaea in the phylogenetic radiation of obligate methylotrophic methanogens, *Methanohalobium* (Zhilina and Zavarzin, 1987) and methane-oxidizing ANME-3 (**Figure 9**; confirmed using cloned nearly full length 16S rRNA sequences (∼1400 bps); see supplementary data for details, Figure S1) almost exclusively dominated sequence reads ascribed to *Methanosarcinaceae* (**Figure 7C**).

**Table 4:** Spearman correlations between depth-wise relative abundance of 16S rRNA genes of specific microbial populations and geochemical parameters.

	Dissolved Fe	Sulfate	JS1	*Desulfobac-teraceae*	ANME-2c	ANME-1	*Methanomi-crobiales*	*Methanosae-cvtaceae*	*Methanohalobium*/ANME-3-related archaea
Dissolved Fe	1.0000	0.8717	**0.0048**	0.6228	0.3046	0.2080	0.2080	**0.0048**	0.1108
Sulfate	-0.0857	1.0000	0.7872	0.1108	0.0341	0.7872	0.7872	0.7872	0.8717
JS1	0.9429	-0.1429	1.0000	0.3965	0.3046	0.1108	0.3287	**0.0000**	0.2657
*Desulfobacteraceae*	-0.2571	0.7143	-0.4286	1.0000	0.3046	0.1108	0.7872	0.3965	0.6228
ANME-2c	-0.5071	0.8452	-0.5071	0.5071	1.0000	0.7489	0.2678	0.3046	0.4679
ANME-1	-0.6000	0.1429	-0.7143	0.7143	0.1690	1.0000	0.7040	0.1108	0.8717
*Methanomicrobiales*	0.6000	-0.1429	0.4857	0.1429	-0.5409	0.2000	1.0000	0.3287	**0.0048**
*Methanosaetaceae*	0.9429	-0.1429	1.0000	-0.4286	-0.5071	-0.7143	0.4857	1.0000	0.2657
*Methanohalobium*/	0.7143	0.0857	0.5429	0.2571	-0.3719	0.0857	0.9429	0.5429	1.0000
ANME-3-related archaea									

*p-values are the values given on right of the “1.000” (in red) diagonal, while the correlation coefficients are displayed to the left. Significant correlations (*p* < 0.05) are the values printed in bold*.

## Discussion

The observation of elevated concentrations of dissolved iron in the methanic zone of marine sediments has been a matter of interest in subsurface geomicrobiology for some time, and it is still not known which microbial population might be involved in the reduction of iron. Here, we find that the distribution of members of the JS1 candidate division, methanogens, and *Methanohalobium*/ANME-3-related archaea co-varies with the profile of pore-water iron, which suggests that these microorganisms might be involved in iron cycling in the methanic zone of the Helgoland mud area.

A number of mechanisms of iron cycling in the methanic zone of marine sediments are discussed till date. One possibility is cryptic sulfur cycling, involving a chemical reaction of downward-diffusing sulfide from the SMT with buried iron (III) minerals, as suggested for Aarhus Bay sediments (Holmkvist et al., 2011). However, in the Helgoland mud sediments this explanation is highly unlikely, because the “sulfidization” front (∼75 cm depths bsf) is too distant from the depths of maximum pore-water iron concentrations. Sulfide is completely depleted at 75 cm depths while the maximum of pore-water iron occurs between 275 and 350 cm (**Figure 2B**). This suggests that a different mechanism might be responsible for iron reduction observed below the SMT.

Dissimilatory iron reducers such as members of the *Desulfuromonadales* are present in the methanic zone and are potential contributors to organoclastic or hydrogenotrophic reduction of iron (III) minerals therein. Unlike in the surface sediments, where they may contribute largely to iron reduction (**Figure 2A**), their relative abundance is low in deeper sediments (**Figure 5**). Based on qPCR-derived bacterial gene copies (10^7^ gene copies per gram wet sediment; **Figure 4**) and their pyrosequencing-derived relative abundances in the methanic zone (0.01–0.1% of total bacteria; **Figure 5**), they were estimated to account for about 10^3^–10^4^ gene copies per gram wet sediment at the depths sampled. Assuming those cells would be viable and active *in situ*, their contribution to iron reduction in the methanic zone of our study site cannot be disregarded considering the relatively large amounts of reactive iron minerals such as lepidocrocite as revealed by Mössbauer spectroscopy (Figures **Figure 3B,C**) and sequential iron extraction (**Figure 3A**) that have been deposited during periods of higher sedimentation (750–1000 years ago) and the absence of free sulfide at the depths below 75 cm. These conditions could allow accumulation of copious amounts of dissolved iron even if iron reduction had occurred at low rates. Nevertheless, the distribution of *Desulfuromonadales* did not correspond to the dissolved iron profiles and are thus most likely these bacteria were not the main organisms reducing iron minerals in the methanic zone.

In the deeper subsurface sediment, the distribution of candidate division JS1 (SB45 lineage) bacteria, however, co-varies with the dissolved iron profile and their direct or indirect linkage to iron reduction seems likely. The main driver of this covariance, Helgoland_JS1_A OTU, is closely related to clone HB2-8-29 (DQ334649), which originated from an industrial harbor basin contaminated with heavy metals and where iron reduction was the dominant metal transformation process (Toes et al., 2008; **Figure 8**).

JS1 populations have previously been found in many methane-containing sediments: in methanic subsurface sediments (Webster et al., 2004, 2007; Parkes et al., 2005, 2007), methane seeps and gas hydrate sites (Inagaki et al., 2006; Siegert et al., 2011b; Chevalier et al., 2013; Lee et al., 2013), and mud volcanoes (Pachiadaki et al., 2011). However, there exists only little knowledge regarding the metabolism of these bacteria. JS1 bacteria appear to be heterotrophic based on their ability to utilize acetate and glucose under low sulfate conditions as revealed by stable isotope probing and enrichment studies (Webster et al., 2006, 2011). Emerging lines of evidence from single cell genomics have also predicted a saccharolytic and fermentative lifestyle for some bacteria in the OP9/JS1 lineage (Dodsworth et al., 2013). As evident from the observed depth-wise increase in ammonium concentrations (**Figure 2B**), degradation of organic matter seems to occur in subsurface sediments of the Helgoland mud area. Members of the JS1 (SB45) lineage have been shown to persist in a sulfate-reducing benzene-mineralizing enrichment culture over 3 years (Phelps et al., 1998). Close relatives of the major JS1 (SB45) member detected in the sediments of our study site (Helgoland_JS1_A), have been found in hydrocarbon-contaminated environments (**Figure 8**). For example, North Sea sediments from which clone HB2-8-29 (DQ334649) was retrieved were contaminated with polychlorinated biphenyl and polyaromatic hydrocarbons (Toes et al., 2008). Clone 2_68_H10-1_b (JQ086866; **Figure 8**) was also detected in hydrocarbon contaminated aquifers (Tischer et al., 2013). One could speculate that some members of the JS1 group may generally play a role in the degradation of hydrocarbon compounds. Moreover, specific members of both JS1 and the *Methanosarcinales* appear to co-exist in marine sediments (Mediterranean Sea, Gulf of Mexico) based on a co-occurrence survey (Chaffron et al., 2010). In the same vein, co-occurrence networks between JS1 bacteria and members of the *Methanomicrobiales* were detected (Gies et al., 2014). The covariance of members of the JS1 bacteria (Helgoland_JS1_A) and specific *Methanosaetaceae* populations (Helgoland_meth3) observed in the sediments studied here (Figures **Figure 7A,B**) suggests that certain members of JS1 bacteria and methanogens interact metabolically possibly via the anaerobic microbial food chain (Schink, 1997).

It has been suggested that AOM may be coupled to the reduction of metal oxides in several aquatic environments: for example, in marine sediments (Beal et al., 2009; Wankel et al., 2012; Egger et al., 2014; Riedinger et al., 2014), freshwater/limnic sediments (Sivan et al., 2011; Segarra et al., 2013). Methanotrophic archaea (ANME) populations are known to be involved in the mediation of anaerobic methane oxidation (Hinrichs et al., 1999; Boetius et al., 2000). The detection of ANME-1, ANME-2c, and uncultured *Methanosarcinaceae* (**Figure 6**), which are mostly related to *Methanohalobium*/ANME-3 (**Figure 7C**), suggests a potential for AOM at the SMT and within the methanic zone of the Helgoland mud area. While the distribution of ANME-1 and ANME-2c populations over depth did not match the dissolved iron profile, *Methanohalobium*/ANME-3-related sequences were highly abundant around the peak of dissolved iron (Figures **Figure 6** and **Figure 7C**). Thus, anaerobic oxidation of methane or methylated one-carbon compounds may be coupled to iron reduction in the methanic zone of our study site. The involvement of ANME-3-related populations in metal reduction in incubations with sediments from the Eel River Basin has been previously suggested by Beal et al. (2009).

Similar depth-wise distribution profiles of *Methanomicrobiales*, *Methanosaetaceae*, and *Methanohalobium*/ANME-3-related *Methanosarcinaceae* (Helgoland_Meth7; **Figure 9**) hint at a possible co-occurrence of methanogenesis and anaerobic oxidation of methane in subsurface sediments of the Helgoland mud area. Evidence for co-occurrence of both processes has been recently reported in sediments from the Bothnian Sea, a site also characterized by the deposition of high amounts of organic matter, non-sulfidic and high dissolved iron in the methanic zone (Egger et al., 2014). However, microorganisms have not been identified from this site yet. *Methanomicrobiales* and *Methanosaetaceae* harbor obligate hydrogenotrophic and acetotrophic methanogens, respectively. Some methanogenic archaea are capable of coupling hydrogen and acetate oxidation directly to iron reduction in pure culture (Bond and Lovley, 2002; van Bodegom et al., 2004; Liu et al., 2011; Zhang et al., 2012; Yamada et al., 2014). Addition of ferrihydrite as a potential electron acceptor increased *mcrA* gene copy numbers of methanogens in incubations with heavy metal- and hydrocarbon-contaminated mud sediments from Zeebrugge harbor basin (Siegert et al., 2011a). Transient conservation of energy from ferrihydrite reduction by Rice Cluster I methanogens from rice field soils has also been suggested (Lueders and Friedrich, 2002). In the presence of high amounts of reactive iron minerals, methanogenesis can be directly inhibited as electrons are diverted to iron reduction (Bond and Lovley, 2002; van Bodegom et al., 2004). The Helgoland mud area is characterized by high sedimentation rates (Hebbeln et al., 2003) and consequently high burial rates of organic matter and iron minerals. Such conditions may cause some of the electrons for methanogenesis to be shunted to oxidized iron minerals implicating methanogenic archaea to be involved in iron reduction in the subsurface sediments of our study site.

## Conclusion

Although the actual mechanisms guiding the interplay of JS1 metabolism, iron reduction, methanogenic, and ANME-3-related archaea activities are not yet clear, our results suggest a close relationship amongst members of the JS1 bacteria, specific methanogens and *Methanohalobium*/ANME-3-related archaea. These associations may also have an influence on iron cycling in subsurface sediments of the Helgoland mud area and other high-accumulation depositional environments such as the Amazon Fan (Flood et al., 1995; Kasten et al., 1998), Argentine Basin (Hensen et al., 2003; Riedinger et al., 2005, 2014), Zambesi Fan (März et al., 2008), and Bothnian Sea (Slomp et al., 2013; Egger et al., 2014), where high sedimentation rates enable the preservation and burial of reactive iron oxides to greater depths. In addition, our results add to the evidences that microorganisms are important in shaping the geochemical environment in sub-seafloor sediments.

## Conflict of Interest Statement

The authors declare that the research was conducted in the absence of any commercial or financial relationships that could be construed as a potential conflict of interest.
