# Supplementary material for: Distinct microbial populations are tightly linked to the profile of dissolved iron in the methanic sediments of the Helgoland mud area, North Sea
Source: Front Microbiol. 2015 May 1;6:365. doi: 10.3389/fmicb.2015.00365 (PMC4416451; doi:10.3389/fmicb.2015.00365)
Supplement: Supplementary file 1 [file Data_Sheet_1.DOCX]

***Supplementary Material***

**Distinct microbial populations are tightly linked to the profile of dissolved iron in the methanic sediments of the Helgoland mud area, North Sea**

Oluwatobi Oni^1,2^, Tetsuro Miyatake^1^, Sabine Kasten^2,3^, Tim Richter-Heitmann^1^, David Fischer^2,3^, Laura Wagenknecht^3^, Ajinkya Kulkarni^1^, Mathias Blumers^4^, Sergii I. Shylin^4,6^,Vadim Ksenofontov^4^, Benilde F.O. Costa^5^, Göstar Klingelhöfer^4^, Michael W. Friedrich^1,2^

1. Microbial Ecophysiology group, Faculty of Biology/Chemistry, University of Bremen, Bremen, Germany
2. MARUM, Center for Marine Environmental Sciences, University of Bremen, Bremen, Germany
3. Alfred Wegener Institute Helmholtz Center for Polar and Marine Research, Bremerhaven, Germany
4. Institute for Inorganic and Analytical Chemistry, Johannes Gutenberg University, Mainz, Germany
5. CFisUC, Physics Department, University of Coimbra, Coimbra, Portugal
6. Department of Chemistry, Taras Shevchenko National University of Kyiv, Volodymyrska, 64/13, 01601 Kyiv, Ukraine

Corresponding author:

Michael W. Friedrich

Address: Microbial Ecophysiology group, Faculty of Biology/Chemistry, University of Bremen, Leobenerstraße, 28359, Bremen, Germany

E-mail: michael.friedrich@uni-bremen.de, Phone: +49-421-218-63060

1. **SUPPLEMENTARY METHOD**

**ARCHAEAL 16S rRNA GENE CLONING AND SEQUENCING**

Cloning was done to obtain long archaeal 16S rRNA genes (ca. 1.3 to 1.4 kbps) in order to validate the phylogenetic placement of methanogen sequences provided in Fig. 9. DNA extract from two depths of the sediment 55 cm - 80 cm and 330 cm - 355 cm (Fe^2+^ maximum) were used for PCR amplification of archaeal 16S rRNA genes with primer pairs Arch109F (5’ ACKGCTCAGTAACACGT 3’) (Großkopf et al., 1998) and Arch1492R (5’ GGCTACCTTGTTACGACTT 3’) (modified from Miyashita et al., 2009)). Purified PCR products were cloned into pGEM-T vector system (Promega, Mannheim, Germany) and positive clones were selected using the blue-white screening assay. 16S rRNA gene fragments were amplified by colony PCR (Subcloning Notebook Guide, BR152,Promega) using plasmid specific M13F-40 (5’ GTTTTCCCAGTCACGAC 3’) and M13b (5’ CAGGAAACAGCTATGAC 3’) primers (Promega) and PCR products were submitted to LGC Genomics (Berlin, Germany) for bi-directional Sanger sequencing using M13 primers. Forward and reverse sequences were merged using SeqMan Pro (DNA Star, version 8.1.2 (33.3), 418) and saved as FASTA files. Clone sequences were aligned using SINA aligner (www.arb-silva.de/aligner) (Pruesse et al., 2007) with the archaeal variability profile. The alignment was imported and curated in ARB version 6.0.2 (Ludwig et al., 2004) using the archaeal SSU filter. Taxonomic identification was done by inserting the aligned sequences into the 16S rRNA gene SILVA non-redundant reference database (SSU Ref NR 99, Version 119 (Quast et al., 2013)) using the ARB Parsimony tool. Sequences belonging to families containing known methanogens were selected and re-aligned along with archaeal OTUs obtained from 454 sequencing using the SINA aligner. The aligned sequences were used to generate a maximum likelihood tree with 200 bootstraps using MEGA 5.2.2 (Tamura et al., 2011) and the General Time Reversal (GTR) substitution model. Archaeal 16S rRNA sequences from clone libraries have been submitted to NCBI GenBank under the accession numbers KP987241- KP987264.

1. **SUPPLEMENTARY RESULT**

**Fig S1**.Maximum likelihood tree of archaeal 16S rRNA gene sequences showing the phylogenetic affiliations of methanogen and ANME-3 sequences from 454 sequencing (Helgoland_meth, ca.200bp sequence length ) and Sanger sequencing (A/AI; ca. 1.3 to 1.4 kbps). All methanogen sequences retained their positions as presented in Fig. 9. Helgoland_meth7 is closely related to *Methanohalobium*/ANME-3. *Clostridium carboxidivorans* (FR733710), *Desulfuromonas svalbardensis* (AY835388) and *Desulfuromonas michiganenesis* (AF357915) serve as outgroups. Bar represents 20% sequence divergence.

**REFERENCES**

Großkopf R., Stubner, S., and Liesack, W. (1998). Novel euryarchaeotal lineages detected on rice roots and in the anoxic bulk soil of flooded rice microcosms. *Appl. Environ. Microbiol.* 64, 4983–9.

Ludwig, W., Strunk, O., Westram, R., Richter, L., Meier, H., Yadhukumar, Buchner, A., Lai, T., Steppi, S., Jobb, G., et al. (2004). ARB: a software environment for sequence data. *Nucleic Acids Res.* 32, 1363–71. doi:10.1093/nar/gkh293.

Miyashita, A., Mochimaru, H., Kazama, H., Ohashi, A., Yamaguchi, T., Nunoura, T., Horikoshi, K., Takai, K., and Imachi, H. (2009). Development of 16S rRNA gene-targeted primers for detection of archaeal anaerobic methanotrophs (ANMEs). *FEMS Microbiol. Lett.* 297, 31–7. doi:10.1111/j.1574-6968.2009.01648.x.

Pruesse, E., Quast, C., Knittel, K., Fuchs, B. M., Ludwig, W., Peplies, J., and Glöckner, F. O. (2007). SILVA: a comprehensive online resource for quality checked and aligned ribosomal RNA sequence data compatible with ARB. *Nucleic Acids Res.* 35, 7188–96. doi:10.1093/nar/gkm864.

Quast, C., Pruesse, E., Yilmaz, P., Gerken, J., Schweer, T., Yarza, P., Peplies, J., and Glöckner, F. O. (2013). The SILVA ribosomal RNA gene database project: improved data processing and web-based tools. *Nucleic Acids Res.* 41, D590–6. doi:10.1093/nar/gks1219.

Tamura, K., Peterson, D., Peterson, N., Stecher, G., Nei, M., and Kumar, S. (2011). MEGA5: molecular evolutionary genetics analysis using maximum likelihood, evolutionary distance, and maximum parsimony methods. *Mol. Biol. Evol.* 28, 2731–9. doi:10.1093/molbev/msr121.
